# The Scoop on SCOBY (Symbiotic Culture of Bacteria and Yeast): Exploring Consumer Behaviours towards a Novel Ice Cream

**DOI:** 10.3390/foods12173152

**Published:** 2023-08-22

**Authors:** Annu Mehta, Luca Serventi, Lokesh Kumar, Damir Dennis Torrico

**Affiliations:** Faculty of Agriculture and Life Sciences, Lincoln University, Lincoln 7647, New Zealand; annu.mehta@lincolnuni.ac.nz (A.M.); luca.serventi@lincoln.ac.nz (L.S.); lokesh.kumar@lincoln.ac.nz (L.K.)

**Keywords:** theory of planned behaviour (TPB), emotions, SCOBY, food neophobia, sensory evaluation, consumer research

## Abstract

With the growing demand for sustainable practises, the food industry is increasingly adopting circular economy approaches. One example is recycling the symbiotic culture of bacteria and yeast (SCOBY) used in kombucha fermentation to create value-added products. However, consumer acceptance of such novel products remains unclear. To address this, the present study examined consumer attitudes towards ice cream made with SCOBY as an ingredient and how this affected their intention to consume it. Drawing on the theory of planned behaviour (TPB) and additional constructs such as emotions and food neophobia, an online survey was conducted with New Zealand consumers (N = 170). Results showed that the TPB constructs significantly predicted the intention to consume SCOBY ice cream. Moreover, by adding emotions to the constructs, the model’s explanatory power was enhanced. Attitudes, subjective norms, and emotions were the main predictors of intention, which in turn was found to be the main predictor of behaviour. Participants’ beliefs about the safety and taste of SCOBY ice cream were significantly correlated with their intention and behaviour, as were the opinions of nutritionists/dietitians, friends, and family. The model accounted for 21.7% of the variance in behaviour and 57.4% of the variance in intention. These findings can be used to plan marketing strategies related to waste-to-value-added products such as SCOBY ice cream.

## 1. Introduction

Waste-to-value foods represent a novel product category that promotes a circular economy and sustainability by converting waste or surplus ingredients into new value-added products [1]. For instance, Kombucha, a popular beverage derived from fermented black tea utilising a Symbiotic Culture of Bacteria and Yeast (SCOBY), generates substantial quantities of SCOBY remnant waste [2,3]. Commercialising this waste could offer a viable approach for transforming this discarded residue into value-added foods while fostering sustainable practises. Moreover, SCOBY, a three-dimensional bacterial cellulose mat formed by the symbiotic relationship between acetic acid bacteria and osmophilic yeast species, has various potential uses and health benefits [4,5,6,7]. Bacterial cellulose possesses properties such as high water absorption, biocompatibility, high mechanical strength, and porosity, making it suitable for applications in edible packaging and food fortification [3]. Moreover, the antibacterial properties of bacterial cellulose make it effective against harmful Gram-positive bacteria such as *Staphylococcus, Bacillus, Pseudomonas*, and *Enterobacter* and Gram-negative bacteria such as *Escherichia coli* [6]. Kombucha has high levels of probiotics, antioxidants, anti-inflammatory, anti-carcinogenic, and anti-diabetic effects [2]. Polyphenols and volatile flavour compounds found in kombucha tea, such as theaflavins, have been shown to control cholesterol levels by inhibiting triglycerides and LDL oxidation [8] and assist in weight management [5,9].

Ice cream is a widely consumed food product that individuals of all ages enjoy. However, ice cream is often perceived as unhealthy due to its high fat and sugar content [10]. Adding SCOBY to ice cream formulations has the potential to yield a novel product with enhanced hedonic, nutritional, and probiotic properties [11,12]. Ice cream, as a dairy product, offers a unique advantage as a carrier for probiotic bacteria. Incorporating probiotic bacteria into ice cream could potentially create an improved, functional, and healthy version of this food. Ice cream contains essential nutrients such as proteins, fats, vitamins, and minerals, further enhancing its nutritional value. Also, compared to fermented milk and yoghurts, ice cream provides a more favourable environment for the viability of probiotic strains during production and storage [13]. In addition to its nutritional benefits, ice cream is enjoyed due to its desirable sensory properties. The creamy texture and sweet taste contribute to its popularity among consumers of all ages, including kids, adolescents, adults, and older adults [14]. However, introducing a novel ingredient into a traditional product could elicit resistance among consumers [15,16,17]. Factors such as consumer behaviour and attitude towards the novel product, food neophobia, and unfamiliarity affect consumers’ product choices [18]. Previous studies have used psychographic factors to predict consumer behaviour and attitudes towards novel products. The Theory of Planned Behaviour (TPB) is one of the theoretical approaches widely used to predict consumer intention and behaviour towards insect food [19,20,21], organic food [22,23], ethnic foods [24], and genetically modified foods [25,26].

Every year, an array of value-added products is launched into the market due to increased consumer demand. However, most new and value-added products launched go unnoticed due to a lack of product knowledge, exposure, and food neophobia. Food neophobia, or the unwillingness to try a new product, significantly affects food choices [18]. Food neophobia, a personality trait characterised by an aversion to new experiences or food, is a key predictor of approach-based behaviour in individuals. The strong influence of food neophobia on consumer attitudes and intentions towards novel products has been reported earlier [27,28,29]. Therefore, it is crucial to examine its effect in this context. Emotion is another important factor that significantly affects decision-making [30]. The role of emotions as a motivator of actions and choices has been extensively studied [31]. In the highly competitive market for newly developed products, emotional measurements can provide a broader perspective of the consumer’s experiences and complement the measures of sensory acceptability (liking) regarding value-added products [32].

Hence, the main objective of the present study was to understand the relevant variables that predict the consumer’s willingness to try SCOBY ice cream as a value-added product based on the theory of planned behaviour. The additional variables, emotions and food neophobia, were added to the original model to increase the predictive power regarding consumers’ intentions to try SCOBY ice cream. The findings will help understand the consumer’s behaviour and purchase intention to improve marketing strategies for waste-to-value-added products.

## 2. Theoretical Background

### 2.1. Theory of Planned Behaviour (TPB)

The theory of planned behaviour (TPB) is widely used to interpret the driving factors influencing human behaviour [33]. The TPB suggests that behaviour is directed by intention (willingness to try), which is further guided by attitude (positive or negative appraisals of behaviour), subjective norm (social pressure to perform or not perform the behaviour), and perceived behavioural control (individual ability to perform the behaviour). The TPB model has been successfully used in food-related studies [34] to predict consumers’ intentions to try and buy novel food products such as organic foods [23], insect-based foods [19], game meats [35], fast foods [36], 3D-printed foods [37], plant-based foods [38], and halal meats [39]. The TPB model is believed to be a strong evidence-based predictor of food choices. Based on previous food-related behaviour studies, the predictive power of TPB varies from 50% to 70% for intention prediction and ranges from 20% to 40% for behaviour prediction [40].

#### 2.1.1. Intention and Willingness

Intention, which reflects cognitive planning and motivational factors, is commonly used to predict behaviour. Willingness is an unintentional and irrational factor influencing behaviour and can also play a significant role in behaviour prediction [40]. Despite its importance, limited research has been conducted on the role of willingness in shaping behaviour. Moreover, intention is a reliable predictor of approach-based behaviour towards new food categories, such as game meats [41] and insect cookies [42].

#### 2.1.2. Attitude

Attitude refers to motivational values towards or away from behaviour [33]. Attitude is the individual core beliefs formed from belief strength and its outcome evaluation. TPB postulates that attitude towards behaviour is a function of opinions and their outcomes (positive or negative). For example, studies have shown that attitudes towards novel products such as game meats [41], hemp food [43], and cultured meats [44] affect the consumer’s purchase intention.

#### 2.1.3. Subjective Norm

Ajzen (1991) stated that subjective norms refer to the pressure individuals experience from their social environment, including friends, family, or colleagues, which can influence their decision-making [33]. Subjective norms incorporate both injunctive and descriptive norms [40]. The injunctive norm reflects what others want an individual to do, while the descriptive norm reflects the individual’s perception of whether others engage in the behaviour. In this study, only injunctive norms were included, as SCOBY ice cream is new to the New Zealand market and few people know the benefits of SCOBY and kombucha. Injunctive norms have a more significant impact when asked to indicate a consumer’s intention to make a food choice in the future, as they are not affected by visceral factors such as hunger, mood, and emotions, which is expected in the case of descriptive norms [45]. For each injunctive norm, the motivation to comply with the beliefs of others was also determined. Understanding social norms is important in the study of food behaviours, as previous studies have shown that these factors have positively influenced the willingness to try and purchase intention of halal meats [39] and game meats [41].

#### 2.1.4. Perceived Behaviour Control (PBC)

Perceived behaviour control (PBC) is a construct that refers to an individual’s perception of ease or difficulty in performing a particular behaviour. Control beliefs can either facilitate or impede the behaviour of interest. The more resources and opportunities individuals have, the fewer chances of impediment; hence, a stronger effect of PBC on behaviour will be exhibited [33]. PBC has successfully predicted the behaviour and intention of consumers towards novel foods such as insect-based cookies [42], hemp food [43], and in vitro meats [46]. Based on the primary constructs of TPB, the following hypotheses have been proposed:

**H1.**  *The intention would predict the future behaviour of consuming SCOBY ice cream*.

**H2.**  *The TPB construct attitude predict the intention to try SCOBY ice cream*.

**H3.**  *The TPB construct subjective norm predict the intention to try SCOBY ice cream*.

**H4.**  *The TPB construct PBC predict the intention to try SCOBY ice cream*.

**H4a.**  *Perceived behaviour control can predict the future behaviour of consuming SCOBY ice cream*.

### 2.2. Additional Constructs

#### 2.2.1. Emotions

Emotions are non-conscious appraisals and action tendencies strongly influencing consumer behaviours and decision-making processes, including attitudes and intentions towards accepting value-added products in the market. Various methods have been developed to measure emotions: physiological (magnetic resonance imaging (MRI), electrocardiography (ECG), and electroencephalography (EEG)) [47,48], behavioural (facial expression, voice tone and pitch, and body and posture movements) [29,49,50], and cognitive (self-reported emotions) [29,51]. However, emotions are not a homogeneous category since their multifaceted functions are at work during decision-making. The four-function framework of emotions proposed by Pfister and Böhm (2008) offers a comprehensive approach to categorising emotions and directs strategic decision-making [30]. The four key functions: information (level of pleasantness and unpleasantness associated with the action), speed (affective reaction to advantageous and disadvantageous stimuli), relevance (importance and personal significance of behaviour), and commitment (moral sentiments and pro-social choices) contribute to the decision-making process and influence behavioural outcomes. Limited research has been performed to understand the influence of emotions on human behaviour towards novel upcycled food products. On the other hand, previous studies have examined the direct and indirect effects of emotions on attitudes towards insect-based foods [52], 3D-printed foods [37,53], cultured meats [38,54], genetically modified foods [25,55], and various functional foods [56]. Emotions are the essential antecedents of attitudes and intentions when purchasing novel products. Furthermore, including emotional appraisals in the TPB could enhance the model’s predictive power. In this regard, the positive and negative emotions elicited during the food appraisal could motivate goal-oriented intention [57] and should be used as an independent construct in the model [58]. Based on the role of emotions in the TPB, the following hypotheses have been formulated to support the model:

**H5.**  *Emotions positively influence the intention to consume SCOBY ice cream*.

**H5a.**  *Emotions will positively influence the behaviour control to consume SCOBY ice cream*.

#### 2.2.2. Food Neophobia

Food neophobia is a personality trait that influences a consumer’s acceptance of new products launched on the market. Individuals with this trait are often hesitant to try unknown and novel foods due to different fears, such as the transmission of allergens and/or the presence of pathogens or toxins [59]. The Food Neophobia Scale (FNS) [60], the Food Attitude Survey (FAS) [61], and the Food Technology Neophobia Scale (FTNS) [62] are widely used methods to measure food neophobia. FNS scores are highly predictive of behavioural responses and widely used to measure general food neophobia, trait anxiety, and acceptance of novel and unfamiliar foods [63], such as cultured meats [27,64], insect-based foods [19,65,66], edible seaweeds [28,67], non-traditional foods, and ethnic foods [24,68]. Accordingly, the following hypotheses related to food neophobia have been formulated in support of the TPB model:

**H6.**  *Food neophobia significantly affects the intention to consume SCOBY ice cream*.

**H6a.**  *Food neophobia can predict the consumer’s behaviour towards SCOBY ice cream*.

## 3. Materials and Methods

### 3.1. Research Framework

**Figure 1:**
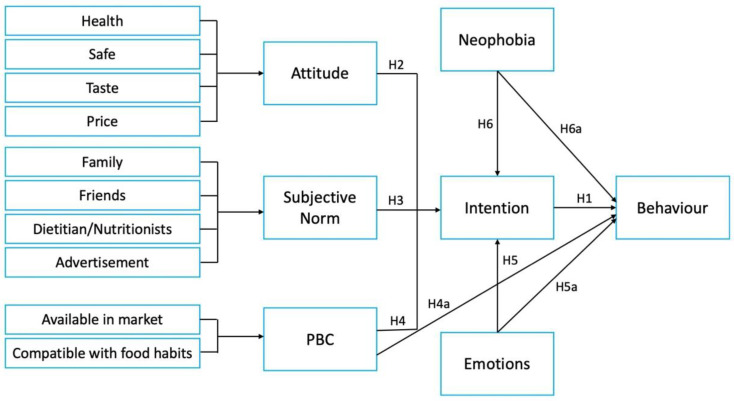
Conceptual framework and hypotheses of this study *. * H1 to H6 are the research hypotheses of this study.

This study was focused on the TPB framework and two additional variables—food neophobia and emotions. In addition, the research evaluated consumers’ attitudes and intentions to try and purchase SCOBY ice cream. Figure 1 shows the research framework of this study.

### 3.2. Experimental Design

#### 3.2.1. Pre-Test

The Lincoln University Human Research Ethics Committee (HEC2021-08) granted ethical approval for this study. The survey was conducted through a questionnaire launched in April 2022 in Lincoln, New Zealand. A pre-test of the measurement scale was performed with thirty-four students at Lincoln University to check the consistency of the indirect questions used to predict the TPB measurements (attitude, subjective norms, and perceived behaviour control), food neophobia, and emotions [69]. In 1996, Bruner and Hensel stated that the high correlation between direct and indirect measurements within the TPB signifies that using indirect questions is a good approach for assessing the model’s underlying construct [70]. To avoid the potential halo effect, selected questions in the pre-test were subject to reverse scaling. The halo effect is the cognitive bias in impression formation, such as carrying over from one judgement to another during data collection [71]. The unipolar and bipolar 7-point Likert scales were used to measure the responses. The Inappropriate statements (based on the pre-test) were modified for the final questionnaire based on reliability tests, Cronbach alpha value, composite reliability, and average variance estimate.

#### 3.2.2. Questionnaire Design

The questionnaire design for the research was divided into four sections (Table S1). The first section provided detailed information regarding SCOBY, its nutritional importance, and a picture of the product. The second section addressed statements related to primary constructs (attitude, subjective norm, and PBC) and their indirect measurements (behavioural, normative, and control beliefs). The third section addressed the statements about food neophobia and emotion towards SCOBY ice cream. Finally, in the last section, demographic information such as gender, age, education, and ethnicity of the respondents was reported.

#### 3.2.3. Measurements

The theory of planned behaviour postulates three direct constructs (attitude, subjective norm, and perceived behaviour control) to predict intention (Table S1). The first construct attitude towards the behaviour (*A*) was measured with four semantic statements using a 7-point unipolar scale: “Consuming SCOBY ice cream in the future will be: not at all pleasant/pleasant, not at all tasty/tasty, not at all relevant/relevant, and not at all important/important” [42]. *Behavioural belief strength* (*b*) is the salient predictor of attitude towards behaviour. It was measured with a 7-point bipolar scale ranging from “strongly disagree” to “strongly agree” using four statements: “Consuming SCOBY ice cream in the future will be: healthy, safe, tasty and expensive”. The *outcome evaluation* (*e*) of each belief strength was measured on a 7-point bipolar importance scale. A belief index is directly proportional to the attitude towards the behaviour, which was determined by Equation (1) [33].

(1) $$A\propto \sum_{i=1}^{n}\left(b_{i}e_{i}\right)$$

The second predictor of intention is the subjective norm [33], also referred to as social pressure. The subjective norm responses were reported on a 7-point bipolar scale (from “strongly disagree” to “strongly agree”): “People who are important to me will approve that I should try SCOBY ice cream”, “People who are important to me will buy SCOBY ice cream” and “People who are important to me supports buying SCOBY ice cream”. Normative beliefs reflect the approval or disapproval of behaviour by important people or groups. It was included in this study as an indirect measure of subjective norm because ice cream made from SCOBY is a novel product and consumers have minimal knowledge about it. Based on a previous study, this study considered three referents: friends, family, and nutritionists/dietitians [42]. An additional referent chosen in this study was an advertisement, as it strongly influences consumers’ opinions and behaviours [72]. The *normative belief strength* (*n*) was measured with a 7-point bipolar scale with statements: “I will consume SCOBY ice cream if my friends/family/nutritionist/advertisement encourages me” and *motivation to comply* (*m*), with the normative belief being measured with a 7-point unipolar scale ranging from “not at all important” to “extremely important”. As mentioned in Equation (2) [33], the subjective norm (*SN*) is directly proportional to the sum of the strength of the normative belief (*n*) multiplied by the motivation to comply (*m*) with each strength across the n number of salient referents.

(2) $$\mathrm{SN}\propto \sum_{i=1}^{n}\left(n_{i}m_{i}\right)$$

Three statements measured the perceived behaviour control: “The decision to consume SCOBY ice cream will be completely under my control”, “The ability to purchase SCOBY ice cream will be entirely up to me” and “I am confident in my decision to buy SCOBY ice cream” on a 7-point bipolar scale. *Control belief* is an indirect measure of PBC. It measures the factors that impede or expedite behaviour, and the *perceived power* (*p*) was measured with a 7-point bipolar scale [33]. Both statements were designed based on the unavailability of SCOBY ice cream in the market and its incompatibility with food habits [42]. Equation (3) depicts the relation between PBC and the sum of the multiplication of control belief (*c*) and perceived power (*p*).

(3) $$\mathrm{PBC}\propto \sum_{i=1}^{n}\left(c_{i}p_{i}\right)$$

The intention towards the behaviour was assessed with two statements: “I intend to eat SCOBY ice cream in the future” and “For sure I will eat SCOBY ice cream in the future” on a 7-point bipolar scale, ranging from “strongly disagree” to “strongly agree”. The confirmation of the *behaviour* was measured on a binomial scale (yes/no will consume the SCOBY ice cream on invitation).

Food neophobia and emotions are two independent constructs added to this study based on their crucial roles in accepting SCOBY ice cream. The statements to measure food neophobia were obtained from the food neophobia scale (range: 10–70) [60]. Based on the four-function framework provided to measure emotions [30,73], four statements related to emotions were formulated: (1) information: “After eating SCOBY ice cream, I will feel (hopeless/hopeful)”; (2) speed: “Consuming SCOBY ice cream will make me feel (discouraged/encouraged)”; (3) relevance: “The thought of consuming SCOBY ice cream makes me feel (unfulfilled/fulfilled)”; and (4) commitment: “I feel guilty/proud when I think of trying SCOBY ice cream” on a 7-point bipolar scale.

The participants were asked about their willingness to perform the behaviour by confirming their participation in the tasting session with dichotomous criteria: “0 = interested in the tasting” and “1 = not interested in the tasting”. In addition, demographic details such as gender, age, education, and ethnicity were also asked at the end of the questionnaire. To confirm their behaviour, two tasting sessions were conducted after a week. During the two tasting sessions, participants received a shorter version of the TPB questionnaire with similar questions to measure their attitude towards SCOBY ice cream and their intention to eat SCOBY ice cream in the future.

### 3.3. Sample Size and Demographic Composition

In this study, the survey forms were distributed to the students and faculty of Lincoln University through email, and 241 forms were returned. After removing 71 incomplete survey forms, N = 170 was used for analysis. Regarding the demographic information, the survey was completed by 69.40% females, 28.24% males, and the rest by non-binary/third-party genders. Based on ethnicity, 32% of respondents were Asian, 39% were white/Caucasian, 19% were New Zealand Europeans, 3% were South Americans, and the rest were African, Māori, and Middle Eastern. Most respondents were 25–54 years old (75.29%), 17.06% were above 55, and 7.65% were between 18 and 24 years old. A total of 86% of the respondents have education at the university level (Bachelor, Master, and Doctoral), and the rest are from the high school level and miscellaneous.

### 3.4. Statistical Analysis

The structural model of this study was evaluated, and the proposed hypotheses were examined to specify the important determinants influencing the intention to purchase SCOBY ice cream. Analysis was conducted using the Statistical Package for Social Sciences SPSS version 29.0.0.0 statistical software (IBM^®^ Corp: New York, NY, USA) and SPSS Amos 28.0.1 (IBM^®^ Corp: New York, NY, USA).

Cronbach’s alpha (C_a_) was used to check the internal consistency of the constructs, along with composite reliability (CR) and average variance extracted (AVE). Spearman’s correlation coefficient was used to determine the correlation between intention, behaviour, and the study’s primary and additional constructs. Chi-Square (χ^2^), the Comparative Fit Index (CFI), the Tucker–Lewis Index (TLI), and the Root Mean Square Error of Approximation (RMSEA) were used to assess the fitness of the model [74]. The coefficient of determination was measured to evaluate the variance of the primary constructs: intention and behaviour. The data were also analysed to measure the correlation between the salient beliefs (behaviour, normative, and control beliefs) and their respective direct measurements (attitude, subjective norm, and perceived behaviour control) and intentions. The statements used to measure different salient beliefs were analysed using Analysis of Variance (ANOVA). The Tukey *post hoc* test was used to find differences between the statements at a 5% significance level. A point biserial correlation was used to find the relationship between demographic variables and intention/behaviour. The agglomerated hierarchical clustering (AHC) method was used to classify consumers based on their attitude, subjective norm, PBC, and intention towards the acceptability of the novel product. The change in attitude and intention to try SCOBY ice cream between pre- and post-tasting sessions was determined using an ANOVA with a Tukey honest test at a 5% significance level. The dissimilarity was used as a clustering criterion and was analysed based on the Euclidean distance and Ward’s method. XLSTAT^®^ statistical analysis software (version: 2021.3.1, Addinsoft, New York, NY, USA) was used for multivariate analysis.

## 4. Results

### 4.1. Model Reliability and Validity

The reliability and model validity values of the construct are shown in Table 1A. The Cronbach’s α values for the latent variables in this study varied from 0.76 to 0.95. Cronbach’s α values higher than 0.70 represent higher reliability, and values lower than 0.35 represent low reliability [75]. The composite reliability of the latent variable ranged from 0.73 to 0.88 in this study. The composite reliability of the latent variables should be greater than 0.70 to confirm the validity of the examined variables used to measure the latent variables [76,77]. Similarly, the average variance extracted for each variable was higher than the recommended benchmark of 0.50, showing high reliability in the results [75].

The means, standard deviations, and correlations between the constructs are shown in Table 1B. Significant positive correlations were found between intention and willingness to try (*r* = 0.39, *p* < 0.01), attitude and willingness to try (*r* = 0.23, *p* < 0.01), subjective norm and willingness to try (*r* = 0.23, *p* < 0.01), PBC and willingness to try (*r* = 0.24, *p* < 0.01), and emotions and willingness to try (*r* = 0.29, *p* < 0.01). Hence, the results show that the higher the intention, attitude, subjective norms, PBC, and emotions elicited by consumers, the higher their chances are to try SCOBY ice cream. Positive attitude (*r* = 0.71, *p* < 0.01), subjective norm (*r* = 0.47, *p* < 0.01), and emotions (*r* = 0.62, *p* < 0.01) increased the intention to try SCOBY ice cream. Furthermore, a significant positive correlation was found between subjective norms and attitude (*r* = 0.44, *p* < 0.01), which indicates that the higher the social pressure to perform the behaviour, the more positive the attitude towards the product will be among the consumers.

### 4.2. Structural Model and Hypothesis Testing

The TPB model had a higher fit of the data [confirmatory data analysis (CFA): χ^2^ (32) = 81.61, comparative fit index (CFI) = 0.96, normalised fit index (NFI) = 0.94, Tucker–Lewis’s index (TLI) = 0.90, and root mean square error of approximation = 0.001]. The recommended values of CFI and TLI should be above 0.90, and RMSEA should be below 0.08 [78]. Based on the coefficient of determination (*R*^2^), the TPB model explained 21.7% and 57.4% of the variance in behaviour and intention to try SCOBY ice cream, respectively (Table 2). Attitude is significantly correlated to intention (*r* = 0.54, *p* < 0.01), which means that the more positive the attitude towards the behaviour, the higher the intention to perform the behaviour. The significant predictors of intention were attitude (β = 0.54, *p* < 0.01), subjective norm (β = 0.16, *p* < 0.01) and emotions (β = 0.20, *p* < 0.01), confirming H2, H3 and H5. In the case of behaviour (willingness to try the SCOBY ice cream), intention (β = 0.33, *p* < 0.01) was the main predictor, followed by PBC (β = 0.17, *p* < 0.01) in this study; hence, proving the confirmation of H1. However, food neophobia was not a significant predictor of intention and behaviour, thus disconfirming H6 and H6a. Similarly, the tested emotions did not significantly predict behaviour (disconfirming H5a).

### 4.3. Salient Beliefs

The salient beliefs of the TPB model, such as behavioural, normative, and control beliefs, influence attitude, subjective norm, and PBC. Their effects further influence the intention and willingness to try SCOBY ice cream [79]. The mean values of belief strength and their outcome evaluation predicting behavioural, normative, and control beliefs are shown in Table 3A. Regarding behavioural beliefs, the participants believed that trying SCOBY ice cream would be safe, but they were not sure about the taste of the ice cream. However, the participants agreed that taste is essential to trying the novel product, followed by safety. Furthermore, nutritionists/dietitians and family recommendations were the main factors that motivated the participants to try SCOBY ice cream, according to normative beliefs. Finally, in controlling beliefs, the availability of the market and compatibility with food habits positively affected behaviours.

The correlation between the salient beliefs, their primary constructs, and the intention to perform the behaviour is shown in Table 3B. In the case of behavioural beliefs, the salient beliefs that have the most significant and sizeable effect on attitude (*r* = 0.58, *p* < 0.01) and intention (*r* = 0.57, *p* < 0.01) were taste. The novel product’s taste is the core factor influencing the attitude and intention to perform the behaviour. The behavioural belief of safety (*safe to try a novel product*) was the next crucial salient belief that significantly influenced attitude (*r* = 0.23, *p* < 0.01) and intention (*r* = 0.24, *p* < 0.01). The health perception of SCOBY ice cream has a significant effect on attitude (*r* = 0.17, *p* < 0.05) but a non-significant effect on intention (*r* = 0.09, *p* >0.05). The salient belief price has a negative but non-significant effect on attitude (*r* = −0.03, *p* >0.05) and intention (*r* = −0.11, *p* >0.05). In this study, all the normative beliefs statistically affect the subjective norm and intention. The opinion of family and friends has the highest effect on the subjective norm (*r* = 0.50 and 0.47, *p* < 0.01) and intention (*r* = 0.45 and 0.41, *p* < 0.01). The opinion of nutritionists/dietitians was least correlated to the subjective norm (*r* = 0.27, *p* < 0.01) but highly correlated to the intention (*r* = 0.41, *p* < 0.01) to try SCOBY ice cream. The control belief of *availability in the market* (*r* = 0.12, *p* >0.05) does not significantly correlate with PBC, while *compatibility with food habits* (*r* = 0.19, *p* < 0.01) positively correlates with PBC. Control beliefs *compatible with food habits* (*r* = 0.26, *p* < 0.01) and *availability in the market* (*r* = 0.17, *p* < 0.01) were significantly and positively correlated with intention.

### 4.4. Hierarchical Cluster Analysis

**Figure 2:**
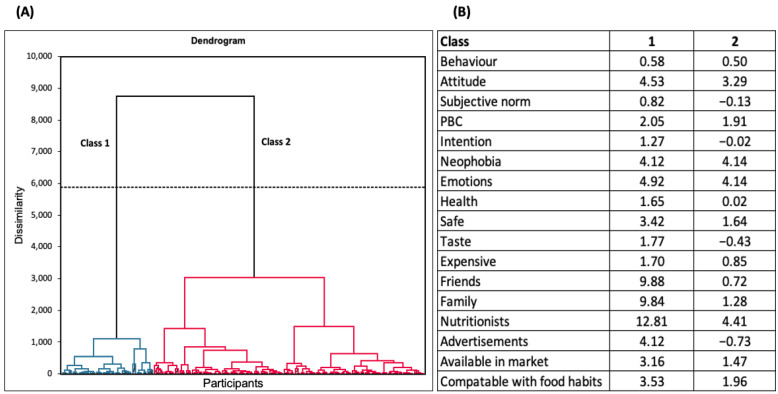
(**A**) Hierarchical cluster analysis (HCA) is used for classifying groups * (Class 1 = blue and Class 2 = red) of participants based on their approach responses. (**B**) Centroids of the two classes * for each factor. * Two class groups were obtained (class 1 (N = 43); class 2 (N = 127)).

The agglomerative hierarchical clustering technique has classified the participants into two classes (class 1 (*N* = 43) and class 2 (*N* = 127)), as shown in Figure 2A. The participants from class 1 (0.58–12.81) strongly agreed with the statements asked in the survey (Figure 2B) compared to class 2 (−0.73–4.41). Classes 1 and 2 were homogeneous concerning gender and education but varied with age and ethnicity (Table 4). Class 1 has a higher proportion of participants in the age group of 35–44 years and below, while class 2 has a higher number of participants in the age group of 45–54 years and above. The classes were also divided based on ethnicity. Class 1 has more participants of Asian ethnicity, while class 2 has more participants of White/Caucasian ethnicity.

### 4.5. Change in Attitude and Intention after Tasting Sessions

**Figure 3:**
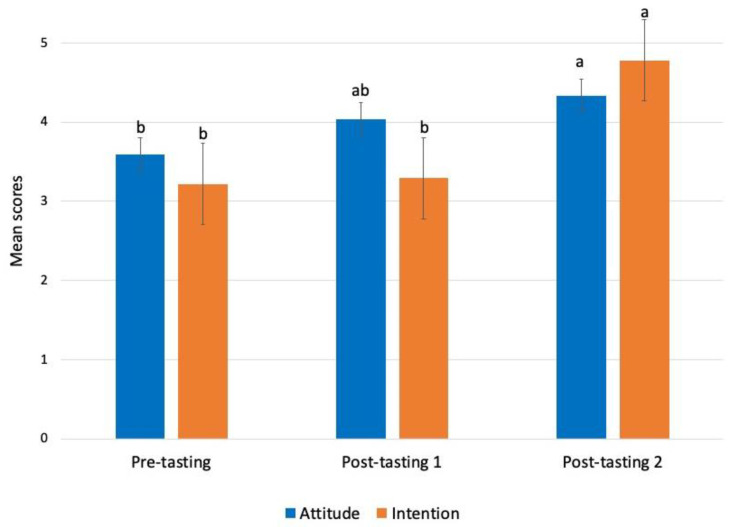
Mean scores of attitudes and intention to eat SCOBY ice cream in pre- and post-tasting sessions. The small alphabet depicts significant difference in attitude and intention between the sessions at a 5% significance level.

The change in attitude and intention after consuming SCOBY ice cream is reported in Figure 3. After the survey, eighty-one participants came for tasting session 1 and session 2 of SCOBY ice cream. Compared to the survey, we found a significant improvement in participants’ attitudes towards consuming SCOBY ice cream in tasting session 1 (4.03, +0.44) and session 2 (4.33, +0.74). The intention to consume the SCOBY ice cream had also significantly increased in tasting session 2 than in the survey (3.22, +1.56) and tasting session 1 (3.29, +1.49). The result showed that consuming a novel product such as SCOBY ice cream multiple times positively affects one’s intention and attitude towards the product.

## 5. Discussion

The present study aimed to identify the constructs that significantly affect the intention to try a SCOBY ice cream based on the extended TPB framework, with emotions and food neophobia as additional predictors of behaviour. The findings suggest that the original constructs of TPB, attitude (54%), and subjective norm (16%) significantly influenced the intention to eat SCOBY ice cream. At the same time, PBC was not an essential factor influencing behavioural intention. The findings were consistent with the previous studies, where attitude was the significant predictor of intention [80,81], followed by the subjective norm. For example, attitudes towards GMO foods (45%) [81], plant-based drinks (22.8%) [1], and organic food (75%) [82] were significant predictors of behavioural intention to buy these products. In this study, the subjective norm strongly predicted intention, while PBC was a non-significant predictor. However, the predictive abilities of PBC and subjective norms varied across the behaviours. Based on the study related to insect flour [42], PBC was a significant predictor of intention, while the subjective norm was a weak predictor. On the other hand, attitude and subjective norms positively influence intention and behaviour towards game meats [41].

SCOBY ice cream is a novel product and is not available commercially. Thus, due to limited knowledge, accessibility, and awareness, the opinions of people important to consumers are more important than the PBC. The participants have emphasised the importance of social norms; therefore, retailers can utilise social norms strategically to influence consumer behaviour, as it does not involve conscious processing [83]. The additional construct’s emotions significantly predicted the intention to try SCOBY ice cream. On the other hand, food neophobia was a non-significant predictor of intention to eat SCOBY ice cream. Ice cream is a hedonic and familiar product, and consumers feel safe when they consume novel ingredients in a familiar food product [84]. Moreover, previous studies have reported that the nutritional enrichment of ice cream is a tested strategy to increase the consumer’s liking and purchase intention [85,86], hence a familiar concept. SCOBY ice cream was prepared using novel food technology, and the Food Technology Neophobia Scale (FTNS) [62] proved to be a highly stable, reliable, and predictive instrument for gauging consumer attitudes and behaviours towards foods created using novel technologies and food fortification compared to the FNS. The high internal consistency (Cronbach alpha = 0.83) and its successful application in previous studies [87,88,89] underscore its status as a dependable psychometric tool for measuring the acceptance of novel food technologies and fortifications for future studies.

Emotions were more relevant than the other determinants of intention. The positive emotions elicited by the SCOBY ice cream played a primary role in shaping consumers’ intentions towards the novel product. Emotions are intense, subconscious appraisals [90], which are always part of all decision-making processes [30] and can also be seen in this study.

Intention, PBC, emotions, and food neophobia explained 21.7% of the behaviour variance, while the TPB model’s primary and additional constructs explained 57.4% of the variance in intention. Previous studies suggest that the TPB can account for a 65–78% variance in intention and a 19–71% variance in behaviour [22,42,43,82,91,92]. However, the predictive power in the studies related to future behaviour is low compared to those on concurrent behaviour [93]. In concurrent behaviour, consumers are familiar with the product. On the other hand, in studies related to future behaviour, the consumers have no exposure to or knowledge about the product, making them reluctant to perform the behaviour. Therefore, consumers should be exposed to novel products before they are launched on the market. As seen in the present study, continuous exposure to SCOBY ice cream improved the attitude and intention to eat it (Figure 3). The other reason for low predictive power can be a lack of familiarity or a delay in performing the behaviour. Bäckström et al. (2003) also reported that the perception of the safety of functional foods due to a lack of familiarity could delay fulfilling the behaviour [94].

TPB’s salient behavioural, normative, and control beliefs have a desired influence on attitude, subjective norm, and perceived control, significantly affecting the intention to perform the behaviour [95]. Based on behavioural beliefs, consumers were more concerned about the safety of the novel product [42]. Similar findings were also reported in other studies, which state that safety and health benefits are the two main factors determining choices in the case of game meats, hemp, and organic foods [41,43,96]. The bacterial cellulose SCOBY is a novel waste to a value-added product whose knowledge was limited to consumers, resulting in concerns about the product’s safety. Therefore, promoting the safety and health benefits of novel products may fortify attitudes, intentions, and behaviours. However, the taste of SCOBY ice cream was essential for consumers. The recommendation of a nutritionist and dietitian, followed by family and friends, was an important intervention for consumers to consider. SCOBY and kombucha have many medicinal properties, such as high levels of probiotics, anti-inflammatory, anti-diabetic, and anti-carcinogenic properties, which makes the opinions of nutritionists and dietitians more relevant to consumers [2,3]. Therefore, the type of product launched and whose opinion can significantly influence the consumers should be considered before undertaking the novel product.

Based on the AHC results, the consumers were divided into two classes based on age and ethnicity. In class 1, the consumers aged 35–44 years (41.86%) had positive attitudes and intentions towards the SCOBY ice cream and expressed less food neophobia towards this product than in class 2. Ethnicity was also significant in predicting the intention to try SCOBY ice cream. Consumers of Asian ethnicity were more accepting of the novel product and expressed less food neophobia than White/Caucasians. Kombucha is of Asian origin and first originated in North-Eastern China [97]. SCOBY and kombucha are widely used for medicinal purposes in many Asian countries, while they are still a new concept in Western countries [98]. Western society has rarely experienced SCOBY as a food source, which can be the reason for a high level of food neophobia and a low attitude and intention to try the product. Similar observations were also reported by Hartmann and Siegrist (2016), who stated that Westerners resist trying insect products due to less exposure and high food neophobia [84]. Hence, the key factors influencing food choices and preferences are demographic variables [99] and product exposure [100].

As mentioned earlier, repetitive exposure to SCOBY ice cream has improved the attitude and intention to eat the novel product. Introducing a novel ingredient (SCOBY) into a familiar product (ice cream) can also enhance the consumer’s familiarity with the novel ingredient. According to Hartmann et al. (2015), introducing insects into familiar food products reduced food neophobia and increased attitudes and willingness to eat insects [20]. Similar results were also reported in the case of other food types, such as different kinds of cheese [101], chillies [102], and fruits and vegetables [100]. The effect of repetitive exposure to the SCOBY ice cream on the sensory perception and liking of the novel product could provide deeper insight into the consumer’s food choices and acceptance.

## 6. Limitations and Future Recommendations

In the present study, certain limitations must be addressed while interpreting the results. First, the findings were based on participants from a pool of students or faculty at the university. Since the sample was selected from this particular group, it may not accurately represent the broader population or the target market for SCOBY ice cream. Further studies with broader population targets are recommended. Moreover, this study focused on consumers’ intentions to consume SCOBY ice cream rather than their actual behaviour. Therefore, future studies are recommended to provide a more robust understanding of consumer behaviours towards SCOBY ice cream and other similar products. To obtain a deeper insight into the consumer’s behaviours, researchers should go beyond intentions and include actual consumer behaviour to validate the relationship between intention and actions. Examining consumer behaviours and attitudes towards novel products over an extended period would explain how consumer perceptions and behaviours evolve.

## 7. Conclusions

This study provides valuable insights into the factors that influence consumer perception of SCOBY ice cream and has significant implications for the future of waste-to-value-added foods. The findings highlight the importance of a positive attitude towards SCOBY ice cream in shaping consumer perceptions and intentions to consume it. Building trust in the safety and taste of the product is crucial to fostering greater consumer acceptance. Moreover, this study also emphasises the significance of social influence in consumer decision-making processes. The opinions of nutritionists/dietitians, friends, and family play a significant role in shaping consumers’ intentions to try novel products such as SCOBY ice cream and should be considered while launching the product in the market. Exposure and knowledge about SCOBY before the tasting session significantly impacted the acceptance of the novel product. Therefore, highlighting the importance of novel products and awareness-building efforts could enhance consumer understanding and familiarity with waste-to-value-added products. Based on our knowledge, no or limited TPB studies have been performed on waste-to-value-added products such as SCOBY; therefore, this paper will provide deeper insight into the determinants that can affect the intention to consume SCOBY ice cream. The recent study fills a gap and contributes to a deeper understanding of consumer behaviour, which helps improve marketing and communication strategies for businesses that operate in waste-to-value-added products such as SCOBY ice cream before launching their product into the market.

## Figures and Tables

**Table 1:** (**A**) Cronbach’s α, composite reliability (CR), and average variance estimate (AVE) values of different constructs. (**B**) Spearman’s correlations between the constructs.

(A) Constructs		Cronbach’s α		CR		AVE	
Attitude		0.89		0.84		0.57	
Subjective norm		0.84		0.86		0.68	
Perceived behaviour control		0.76		0.73		0.50	
Intention		0.95		0.75		0.60	
Food neophobia		0.77		0.84		0.72	
Emotions		0.90		0.88		0.65	
**(B) Constructs**	Intention						
Intention	1.00	Attitude					
Attitude	0.71 *	1.00	PBC				
PBC	0.10	0.10	1.00	Subjective norm			
Subjective norm	0.47 *	0.44 *	0.12	1.00	Food neophobia		
Food neophobia	−0.05	−0.09	0.02	−0.10	1.00	Emotions	
Emotions	0.62 *	0.65 *	0.03	0.38 *	−0.11	1.00	Behaviour
Behaviour	0.39 *	0.23 *	0.24 *	0.23 *	0.09	0.29 *	1.00

* *p* < 0.05.

**Table 2:** Structural equation model: R^2^ and standardised regression coefficient (β) of constructs.

Predictors	R^2^	β
Behaviour	0.23	
Intention		0.33 **
PBC		0.17 *
Emotions		0.10
Food neophobia		0.13
Intention	0.57	
Attitude		0.54 **
Subjective Norm		0.15 **
PBC		−0.01
Emotions		0.19 **
Food neophobia		0.05

* *p* < 0.05; ** *p* < 0.01. Attitude and emotions were measured on a unipolar 7-point Likert scale. Intention, subjective norm, and PBC were measured on a bipolar 7-point Likert scale. The behaviour predictor was measured on a binomial scale. FNS was used to measure food neophobia.

**Table 3:** (**A**) Mean values ^1^ of salient beliefs proposed in behavioural, normative, and control beliefs. (**B**) Correlation between the salient beliefs and their respective parameters (attitude, subjective norms, and PBC) and intention.

(A) Behavioural Beliefs	Belief Strength (b)	Outcome Evaluation (e)			
Healthy	0.81 (1.30) ^b^	−0.17 (1.93) ^d^			
Safe	1.18 (1.12) ^a^	1.67 (1.47) ^b^			
Tasty	0.11 (1.30) ^c^	2.43 (0.75) ^a^			
Expensive	0.81 (1.27) ^b^	1.03 (1.15) ^c^			
Normative beliefs	Belief strength (n)	Motivation to comply (m)			
Friends	0.59 (1.45) ^b^	4.02 (1.62) ^ab^			
Family	0.71 (1.45) ^b^	3.75 (1.50) ^b^			
Nutritionists/dietitian	1.18 (1.34) ^a^	4.31 (1.79) ^a^			
Advertisements	−0.38 (1.56) ^c^	2.52 (1.45) ^c^			
Control Beliefs	Belief strength (c)	Power (p)			
Available in the market	1.61 (1.00) ^a^	1.06 (1.84) ^a^			
Compatible with food habits	1.59 (0.95) ^a^	1.14 (1.36) ^a^			
**(B) Parameters**	**Beliefs**	**Correlation of Beliefs with**			
		**Parameters**		**Intention**	
		**r**	***p***	**r**	***p***
Attitude	Health	0.17	*	0.09	ns
	Safe	0.23	**	0.24	**
	Taste	0.58	**	0.57	**
	Price	−0.03	ns	−0.11	ns
Subjective norm	Friends	0.47	**	0.41	**
	Family	0.50	**	0.45	**
	Nutritionists/dietitian	0.27	**	0.41	**
	Advertisements	0.32	**	0.36	**
PBC	Available in the market	0.12	ns	0.17	*
	Compatible with food habits	0.19	*	0.26	**

^1^ Mean and standard deviation values are measured on a unipolar 7-point scale (ranging from 1 to 7) and the bipolar 7-point scale (ranging from −3 to +3) using analysis of variance (ANOVA) at a 5% significant level. In addition, belief strength (b, n, and c), outcome evaluation (e), and power (p) were measured on the bipolar scale, and motivation to comply (m) was measured on the unipolar scale. Different alphabet in superscript shows a significant difference within the salient beliefs of behavioural, normative, and control beliefs. * r = correlation coefficient. *p* = probability value. *p* < 0.05; ** *p* < 0.01; ns = non-significant.

**Table 4:** Relative frequencies of age and ethnicity for classes 1 and 2.

Class Group ^1^	Age ^2^	Rel. Freq. (%)	Statistical Grouping
Class 1	18–24	11.64	A
	25–34	18.60	A
	35–44	41.86	B
	45–54	13.95	A
	55–64	13.95	A
Class 2	18–24	6.30	A
	25–34	24.41	BC
	35–44	23.62	BC
	45–54	27.56	C
	55–64	11.81	AB
	65+	6.30	A
	Ethnicity		
Class 1	White/Caucasian	25.57	A
	Asian	48.84	B
	Latin American	6.98	A
	NZ European	6.98	A
	Other ^3^	11.63	A
Class 2	White/Caucasian	48.04	C
	Asian	25.20	B
	Latin American	1.57	A
	NZ European	17.32	AB
	Other ^3^	7.87	A

^1^ Class groups were obtained from the hierarchical cluster analysis (HCA; Figure 2). ^2^ Cochran Q test along with Sheskin was performed for multiple pairwise comparisons (5% significance level) of age and ethnicity within each class (class 1 (N = 43); class 2 (N = 127)). ^3^ Others include African, Māori, and Middle Eastern. The capital alphabets signifies significant difference within the class.

## Data Availability

The data presented in this study are available on request from the corresponding author. The data are not publicly available due to the ethical and privacy policies of Lincoln University.

## Supplementary Materials

The following supporting information can be downloaded at: https://www.mdpi.com/article/10.3390/foods12173152/s1. Table S1. Questionnaire design of the study.
