# Supplementary material for: The Scoop on SCOBY (Symbiotic Culture of Bacteria and Yeast): Exploring Consumer Behaviours towards a Novel Ice Cream
Source: Foods. 2023 Aug 22;12(17):3152. doi: 10.3390/foods12173152 (PMC10486441; doi:10.3390/foods12173152)
Supplement: Supplementary file 1 [file foods-12-03152-s001.zip › foods-2467490-supplementary.pdf]

Table S1. Questionnaire design of the study

Sections	Statements
1. SCOBY information	SCOBY is a thick, rubbery and cloudy mat made by a symbiotic relationship between acetic acid bacteria and the osmophilic yeast species. SCOBY is used to ferment green tea or oolong tea to make Kombucha, which is a rich source of organic acids, water-soluble vitamins, proteins, lipids, hydrolytic enzymes, minerals, polyphenols and metabolic products of yeast and bacteria. SCOBY fermented tea has several health benefits, such as high in probiotics, anti-inflammatory, antioxidant activity, anti-diabetics, anti-carcinogenic, and improved liver, immune system and gastrointestinal functions.<br>SCOBY ice cream is a novel product made with SCOBY due to its various health benefits, such as high probiotic count. Considering its health benefits, please answer the following questions.
2. Primary constructs and their predictors	
Attitude	Consuming SCOBY ice cream in the future will be (not at all pleasant/pleasant)<br>Consuming SCOBY ice cream in the future will be (not at all tasty/tasty)<br>Consuming SCOBY ice cream in the future will be (Not at all relevant/relevant)<br>Consuming SCOBY ice cream in the future will be (not at all important/important)
Behavioural belief	Eating SCOBY ice cream in the future will be healthy.<br>Eating SCOBY ice cream in the future will be safe.<br>SCOBY ice cream will have a pleasant taste.<br>Eating SCOBY ice cream in the future will be expensive.
Outcome evaluation	How important do you think are the following aspects when you select an ice cream? Health/Safety/Taste/Price
Subjective norm	People who are important to me will approve that I should try SCOBY ice cream.<br>People who are important to me will buy SCOBY ice cream.<br>People who are important to me support buying SCOBY ice cream.
Normative beliefs	I will eat SCOBY ice cream if my family/friends/nutritionist/advertisement encourages me.
Motivation to comply	How important is the opinion of the following people/institutions for you to decide regarding ice cream?<br>family/friends/nutritionist/advertisement
Perceived behavioural control	The decision to eat SCOBY ice cream will be entirely under my control.
	The ability to purchase SCOBY ice cream will be entirely up to me.<br>I am confident in my decision to buy SCOBY ice cream.
Control beliefs	Eating SCOBY ice cream will be possible if it is available in the market.<br>Eating SCOBY ice cream will be possible if it is compatible with my food habits.
Power factor	Availability in the market makes it easy/difficult for me to try SCOBY ice cream.<br>Compatibility with my food habits makes it easy/difficult for me to try SCOBY ice cream.
Intention	For sure I will eat SCOBY ice cream in the future.<br>I intend to eat SCOBY ice cream in the future.
3. Additional constructs	
Neophobia	I always try new and different products.<br>I am afraid to eat things that I have never eaten before.<br>I will eat almost all types of food.
Emotions	Information: After eating SCOBY ice cream, I will feel _______. (hopeless/hopeful)<br>Speed: Consuming SCOBY ice cream will make me feel _______. (Discouraged/encouraged)<br>Relevance: The thought of consuming SCOBY ice cream makes me feel _______. (unfulfilled/fulfilled)<br>Commitment: I feel (__________) when I think to try SCOBY ice cream. (guilty/proud)
4. Demographics	Age, gender, education and ethnicity
